# Global Prevalence of Microvascular Complications in Children and Adolescents With Type 1 and Type 2 Diabetes: A Systematic Review and Meta-Analysis

**DOI:** 10.1155/pedi/8398194

**Published:** 2025-11-26

**Authors:** Yasmin Ezzatvar, Ignacio Hormazábal-Aguayo, Jacinto Muñoz-Pardeza, Jacqueline Páez-Herrera, Rodrigo Yáñez-Sepúlveda, Antonio García-Hermoso

**Affiliations:** ^1^Department of Nursing, University of València, Valencia, Spain; ^2^Vice-Rectorate for Research and Postgraduate Studies, University of Los Lagos (Universidad de Los Lagos), Osorno, Chile; ^3^Health Sciences Department, Public University of Navarra (UPNA), Pamplona, Spain; ^4^Vice-Rectorate for Research and Postgraduate Studies, University of La Serena (Universidad de La Serena), La Serena, Chile; ^5^Physical Education School, Pontifical Catholic University of Valparaíso (Pontificia Universidad Católica de Valparaíso), Valparaíso, Chile; ^6^Faculty Education and Social Sciences, Andres Bello University (Universidad Andres Bello), Viña del Mar, Chile; ^7^School of Medicine, Espíritu Santo University (Universidad Espíritu Santo, UEES), Samborondón, Ecuador

## Abstract

**Aim:**

To quantify the prevalence of microvascular complications of children and adolescents with type 1 and type 2 diabetes by performing a meta-analysis of observational studies.

**Methods:**

A systematic search in PubMed, EMBASE, and Web of Science was performed from 2000 to August 2025. Studies that reported the prevalence of microvascular complications in children and adolescents with diabetes were included. Study characteristics and prevalence estimates were extracted from each study. Pooled prevalence rates for microvascular complications were calculated using a random-effects model with Freeman-Tukey double arcsine transformation to stabilize variance.

**Results:**

A total of 57 studies were included, comprising 51,819 children and adolescents diagnosed with diabetes (type 1: *n* = 44,150 and type 2: *n* = 7,669), with a mean age of 14.5 and 15.2 years old, respectively. Pooled prevalence of complications in youth with type 1 vs. type 2 diabetes was 22.07% (95% CI: 16.86–27.75) vs. 11.04% (95% CI: 2.73–23.45) for peripheral neuropathy, 31.98% (95% CI: 11.13–57.44) vs. 15.37% (95% CI: 3.09–34.29) for autonomic neuropathy, 13.76% (95% CI: 6.43–23.24) vs. 2.97% (95% CI: 0.00–10.33) for retinopathy, and 13.70% (95% CI: 10.25–17.54) vs. 12.63% (95% CI: 7.99–18.07) for nephropathy, with high heterogeneity across studies and no significant differences between diabetes types. Meta-regression analyses showed no significant associations between complication prevalence and HbA1c, diabetes duration, lipid levels, cohort year, or age.

**Conclusions/Interpretation:**

Microvascular complications affect at least one in 10 youths with diabetes before age 20, with similar prevalence in type 1 and type 2 diabetes. Given the high rates and early onset, routine screening and early intervention are essential for all young people with diabetes to prevent or limit progression of vascular damage, regardless of diabetes type, glycemic stability, or disease duration.

**Summary**

**What is already known about this subject?**

• Microvascular complications such as neuropathy, retinopathy, and nephropathy are well-documented in adults with type 1 and type 2 diabetes.
• Children and adolescents with type 1 and type 2 diabetes are at increasing risk of developing these complications, but prevalence estimates vary widely across studies.
• Traditional risk factors like poor glycemic stability and longer diabetes duration are assumed to contribute to complication risk, though evidence in youth is limited.

**What is the key question?**

• What is the global prevalence of microvascular complications in children and adolescents with type 1 and type 2 diabetes, and to what extent do traditional risk factors (e.g., age, HbA1c, diabetes duration, lipid profile) explain variations in prevalence between diabetes types and across studies?

**What are the new findings?**

• Before age 20, at least 1 in 10 children and adolescents with type 1 or type 2 diabetes present with diabetes-related microvascular complications.
• Microvascular complications are frequent in both types of diabetes, with comparable pooled prevalence estimates across types.
• No significant association was found between study-level factors (mean HbA1c, duration of diabetes, lipid levels, insulin dose) and the prevalence of complications in meta-regression analyses.

**How might this impact on clinical practice in the foreseeable future?**

• Our findings reinforce the need for earlier and more routine screening for microvascular complications in all youth with diabetes, regardless of glycemic stability or diabetes duration, and support the implementation of early interventions to prevent or limit further vascular damage.

## 1. Introduction

The incidence of both type 1 diabetes mellitus [1] and type 2 diabetes mellitus [2] is increasing worldwide among children and adolescents. While type 1 diabetes remains the most common form in this age group, the rising prevalence of pediatric obesity has led to a parallel surge in type 2 diabetes diagnoses [3]. This rising trend puts individual health at risk and will also strain healthcare systems and society, with higher treatment demands and greater economic burdens [4]. With earlier onset of diabetes, these individuals face a longer lifetime exposure to hyperglycemia and associated metabolic disturbances, placing them at a higher risk for chronic complications.

Microvascular complications, including diabetic nephropathy, diabetic retinopathy, and peripheral and autonomic neuropathy, can develop within a few years of diagnosis in youth [5]. The risk of earlier onset, faster progression, and increased severity is particularly notable after puberty in type 1 diabetes and in youth-onset type 2 diabetes [6]. However, microvascular complications are typically asymptomatic in their early stages and often remain undetected until significant damage has occurred [5]. Despite their subtle onset, these complications contribute substantially to long-term morbidity. For instance, retinal damage increases the risk of vision loss, whereas kidney involvement may lead to albuminuria and, if left untreated, progress to end-stage renal disease [5]. Peripheral neuropathy can result in foot ulcers and, in severe cases, amputations [7]. Cardiac autonomic neuropathy (CAN), which impairs involuntary nerve function, is also an independent predictor of cardiovascular mortality [8]. Early detection and prevention are therefore critical, especially given the potential for timely interventions to slow or reverse disease progression. Particularly considering that the presence of one microvascular complication often predicts the development of others [9, 10].

Most existing evidence on the prevalence and risk factors for microvascular complications has been derived from adult populations [11]. In children and adolescents, data remain limited and fragmented, with studies varying in population characteristics, diagnostic criteria, and reported outcomes, and some relying on older cohorts from eras when insulin therapy and diabetes management were not comparable to current standards. While some systematic reviews have reported prevalence estimates, ranging from 4% to 39% for autonomic neuropathy and from 0% to 62% for diabetic peripheral neuropathy in youth with type 1 diabetes [12, 13], 7% for retinopathy and 22% in albuminuria in pediatric type 2 diabetes [14, 15], these have generally focused on single diabetes types or individual complications.

To date, no meta-analysis has examined the prevalence of microvascular complications across both type 1 and type 2 diabetes among children and adolescents, nor compared differences in outcomes between them. A clear understanding of the burden of microvascular complications is essential for identifying at-risk populations and guiding tailored clinical care. Therefore, this systematic review and meta-analysis aimed to estimate the prevalence of microvascular complications in children and adolescents with type 1 and type 2 diabetes separately and to identify potential differences in prevalence between diabetes types. Additionally, we explored whether variation in prevalence across studies can be explained by factors such as HbA1c, diabetes duration, lipid levels, or age.

## 2. Methods

This study is a systematic review and meta-analysis that was conducted following the Preferred Reporting Items for Systematic Reviews and Meta-Analyses (PRISMA) guidelines and recommendations. It was registered prior to conduct the review in the International Prospective Register of Systematic Reviews (PROSPERO, registration number: CRD420251121709). No significant deviations from the protocol were made.

### 2.1. Data Sources and Searches

Two investigators (Yasmin Ezzatvar and Antonio García-Hermoso) independently searched PubMed, EMBASE, and Web of Science for studies written in the English language listed from 2000 to August 2025. The search strategy for each database is detailed in Supporting Information: Table S1. A medical librarian was consulted to review the quality of the search strategy. Reference lists of eligible articles were manually examined for further identification of relevant articles. Any disagreement was resolved by consensus with a third author (Jacinto Muñoz-Pardeza).

### 2.2. Study Selection

After reviewing the title and abstract, two investigators (Yasmin Ezzatvar and Antonio García-Hermoso) systematically assessed the full text of identified articles for eligibility. To be eligible for inclusion in the meta-analysis, studies reporting microvascular complications prevalence in children and adolescents with diabetes were selected, according to the following PECOS criteria: (i) participants: children and adolescents (aged <20 years) diagnosed with type 1 or type 2 diabetes mellitus; (ii) exposure: diabetes diagnosis criteria, diabetes type; (iii) outcomes: microvascular complications (i.e., peripheral and autonomous nerve abnormalities, retinopathy, nephropathy); and (iv) study design: observational studies (cross-sectional, cohort studies, population-based studies, registry-based studies), or baseline data from clinical trials if they report prevalence data on the variables of interest. Studies were excluded if they did not report data regarding the variables of interest and/or reported insufficient information for calculating prevalence rates of microvascular complications. Conflicts over study inclusion were resolved by consensus with a third author (Ignacio Hormazábal-Aguayo).

### 2.3. Data Collection Process and Data Items

Data collection was conducted independently by two investigators (Yasmin Ezzatvar and Antonio García-Hermoso), using an Excel spreadsheet specifically designed for the present study. The following information was extracted from each study that met the selection criteria: (i) study characteristics (first author's name, publication year, study location, study design, sample size, study year, information on source of data); (ii) participants' information (sex, mean age, diabetes duration, mean HbA1C, mean total cholesterol, mean LDL levels, mean HDL levels, insulin dose, number of clinical events); (iii) outcome details (methods of assessment, diagnosis criteria); and (iv) study results (number of cases, prevalence rate). Attempts to obtain missing data by contacting the corresponding authors of the original publications were unsuccessful.

### 2.4. Risk of Bias in Individual Studies

The Joanna Briggs Institute checklist for prevalence studies was used to determine the risk of bias of each study [16]. The checklist comprised 9 items for prevalence studies. Each item of methodological quality was classified as “yes,” “no” or “not reported.”

### 2.5. Summary Measures

We estimated the prevalence of microvascular complications, including peripheral neuropathy, CAN, retinopathy, and nephropathy. All analyses were carried out using STATA (version 16.1, STATA Corporation, College Station, TX). A random-effects model with Freeman-Tukey double arcsine transformation was used to stabilize variance. Prevalence rates were pooled, estimating microvascular complications in children and adolescents with type 1 diabetes or type 2 diabetes. The following conditions were considered when conducting our statistical analyses: (1) meta-analyses were only performed for outcomes that were included in three or more studies; (2) in cases of duplicate publications from the same cohort, only the most recent or the study with the largest sample size was included.

### 2.6. Synthesis of Results

Between-study heterogeneity was evaluated using the *I*^2^ statistic, which estimates the proportion of variability due to true heterogeneity rather than chance. Values of ~25%, 50%, and 75% were interpreted as low, moderate, and high heterogeneity, respectively.

### 2.7. Risk of Bias Across Studies

Publication bias was evaluated using DOI plots and the Luis Furuya-Kanamori (LFK) index [17], when an outcome was reported by at least five studies. The DOI plot provides a visual assessment of asymmetry in study results. An LFK index between −1 and +1 suggests no asymmetry (and thus little evidence of publication bias), values between ±1 and ±2 indicate minor asymmetry, and values beyond ±2 suggest major asymmetry and possible publication bias.

### 2.8. Additional Analysis

Meta-regression analyses were conducted to assess whether study-level variables, including years since diagnosis, mean HbA1c, mean age, study period, total cholesterol, LDL levels, and insulin dose, influenced the prevalence of each outcome when there were at least five studies reporting those variables. Additionally, to examine the effects of each result from each study on the overall findings, results were analyzed with each study deleted from the model once.

## 3. Results

### 3.1. Study Selection

**Figure 1:**
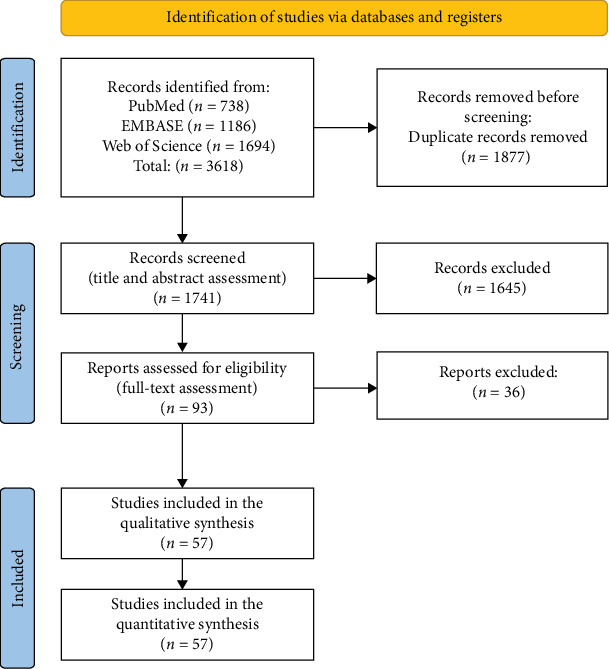
PRISMA flow diagram illustrating the study selection process.

The electronic search strategy retrieved 3618 studies. After removing duplicates and screening titles and abstracts, 96 studies were assessed for eligibility based on full text. A reference list of excluded articles, one along with the rationale for duplicate removal and reasons for exclusion based on full-text review, is provided in Supporting Information: Table S2. The PRISMA flow diagram illustrating the number of studies excluded at each stage of the systematic review and meta-analysis is shown in Figure 1.

### 3.2. Study Characteristics

Characteristics of the 57 included studies [18–75] are summarized in Table 1 (youths with type 1 diabetes) and Table 2 (youths with type 2 diabetes). A total of 51,819 children and adolescents diagnosed with diabetes were included (type 1 diabetes: *n* = 44,150 [42.3% females]; type 2 diabetes: *n* = 7669 [60.6% females]). The mean age was 14.5 ± 2.4 years for those with type 1 diabetes and 15.2 ± 1.5 years for those with type 2 diabetes. The average duration since diagnosis was 6.0 ± 2.1 years for type 1 diabetes and 1.7 ± 0.8 years for type 2 diabetes. Mean HbA1c was 8.8% in type 1 diabetes and 7.8% in type 2 diabetes. The included studies encompassed data from 50 countries across six continents: North America (Canada [38, 65, 66, 73], USA [25, 30, 39, 47, 49, 60, 64, 74]), South America (Brazil [57], Chile [59], Uruguay [45], Venezuela [18]), Europe (UK [29, 72], Germany [53, 63, 68, 69], Denmark [33, 62], Slovakia [55], Sweden [42, 56, 70], Italy [34], Lithuania [21], Austria [20], Greece [46]), Asia (India [23, 27, 28, 71], Iran [24, 54, 58], Turkey [50, 61], South Korea [26, 48], Taiwan [35]), Africa (Sudan [36, 75], Egypt [51, 52], Nigeria [37], Congo [44], Rwanda [43], Tanzania [40, 41]), and Oceania (Australia [22, 32], New Zealand [19, 31]). All studies were published in English and Spanish.

### 3.3. Summary Measures (outcomes)

#### 3.3.1. Peripheral Neuropathy Assessment

Neuropathy was assessed using: (1) nerve conduction studies [20, 24, 27, 28, 48, 75], typically defining neuropathy as abnormalities in conduction velocity or amplitude in at least two nerves; (2) clinical tests such as vibration perception, thermal thresholds, or 10 g monofilaments [22, 33, 40, 51, 54, 56–58, 66, 71]; and (3) standardized scoring systems, most often the Michigan Neuropathy Screening Instrument (MNSI), with cut-offs > 2 [25, 34, 44, 64].

#### 3.3.2. Autonomic Abnormalities Assessment

Most studies assessed CAN, mainly via heart rate variability [30, 50, 64], cardiovascular reflex tests [22, 33, 34, 57], and baroreceptor sensitivity [63]. Studies also assessed autonomic abnormalities with pupillary tests [65], sudomotor function [33, 62], or orthostatic hypotension [41]. Abnormalities were generally defined using age- and sex-matched percentiles or predefined abnormal test counts.

#### 3.3.3. Retinopathy Assessment

Diabetic retinopathy was mainly assessed via fundus photography or ophthalmoscopy [19, 22, 23, 40, 45, 51, 57, 61, 71], using standardized grading systems or clinical examination, with abnormalities defined as at least one microaneurysm or hemorrhage [22, 71] or by graded severity [64].

#### 3.3.4. Nephropathy Assessment

Nephropathy was mainly assessed via albuminuria, typically using the albumin-to-creatinine ratio (ACR) or albumin excretion rate (AER), typically defined as ACR > 30 mg of albumin per gram of creatinine, or AER ≥ 20 *μ* g/min, confirmed in at least two samples [18, 22, 26, 32, 44, 51, 61, 72]. Several studies confirmed results from multiple urine collections over time to confirm persistence [31, 44, 65, 71]. Some studies used 24-h collections [23, 60], spot urine, or clinical records [22, 36, 47], with thresholds occasionally adjusted for age or sex [31, 59, 72]. Broader definitions of diabetic kidney disease included albuminuria ≥ 30 *μ* g/mg and/or reduced estimated glomerular filtration rate (eGFR) ≤ 60 mL/min/1.73 m^2^ [41, 57, 64].

### 3.4. Risk of Bias Within Studies

All studies met at least six out of nine items included in the Joanna Briggs Institute Checklist for prevalence studies and were considered to have fair-to-good methodological quality. The average score was 7.2/9 (Supporting Information: Table S3).

### 3.5. Synthesis of Results

The pooled prevalence estimates of microvascular complications in youth with type 1 and type 2 diabetes are summarized in Table 3. Detailed results of the meta-analyses, including individual forest plots, are provided in the Supporting Information: Figures S1–S4.

#### 3.5.1. Peripheral Neuropathy

The pooled prevalence of peripheral neuropathy was 22.02% (95% CI: (16.86 to 27.75) in youth with type 1 diabetes and 11.04% (95% CI: 2.73–23.45) in those with type 2 diabetes. Heterogeneity was high in both groups (type 1 diabetes: *I*^2^ = 97.91%, *p*  < 0.001; type 2 diabetes: *I*^2^ = 95.86%, *p*  < 0.001), and no significant difference was found between them (*p*=0.104).

#### 3.5.2. Autonomic Neuropathy

The prevalence of autonomic neuropathy was 31.98% (95% CI: 11.13–57.44) in type 1 diabetes and 15.37% (95% CI: 3.09–34.29) in type 2 diabetes. Heterogeneity was high for type 1 diabetes (*I*^2^ = 99.65%, *p*  < 0.001), and no heterogeneity data were available for type 2 diabetes. The between-group difference was not statistically significant (*p*=0.252).

#### 3.5.3. Retinopathy

The pooled prevalence of diabetic retinopathy was 13.76% (95% CI: 6.43–23.24) in type 1 diabetes and 2.97% (95% CI: 0.00–10.33) in type 2 diabetes. Heterogeneity was high in both groups (type 1 diabetes: *I*^2^ = 99.50%, *p*  < 0.001; type 2 diabetes: *I*^2^ = 86.66%, *p*  < 0.001), with no significant difference between them (*p*=0.057).

#### 3.5.4. Nephropathy

The prevalence of nephropathy was 13.70% (95% CI: 10.25–17.54) in type 1 diabetes and 12.63% (95% CI: 7.99–18.07) in type 2 diabetes. Heterogeneity remained high for both (type 1 diabetes: *I*^2^ = 98.44%, *p*  < 0.001; type 2 diabetes: *I*^2^ = 94.51%, *p*  < 0.001), and no statistically significant difference was observed between groups (*p*=0.845).

Subgroup analysis by country income level showed a pooled prevalence of peripheral neuropathy of 24.39% (95% CI 18.27–30.51) in low- and middle-income countries and 23.36% (95% CI 19.45–27.28) in high-income countries, with a significant difference between groups (*p*  < 0.05), as shown in Supporting Information: Figure S5.

### 3.6. Sensitivity Analysis and Publication Bias

The results of the leave-one-out sensitivity analysis showed that omitting any single study did not materially change the pooled effect estimates across outcomes, except for autonomic neuropathy and retinopathy in type 2 diabetes. Detailed information and leave-one-out plots for all outcomes are presented in Supporting Information: Figures S5–S12.

Assessment of publication bias indicated major asymmetry for several outcomes, including peripheral neuropathy in type 1 diabetes (LFK = 4.6), retinopathy in type 1 (LFK = –2.19) and type 2 diabetes (LFK = –2.67), and nephropathy in type 1 (LFK = 6.48) and type 2 diabetes (LFK = 7.64). Minor asymmetry was found for autonomic neuropathy in type 1 diabetes (LFK = –1.93), whereas peripheral neuropathy in type 2 diabetes showed no asymmetry (LFK = –0.95, Supporting Information: Figures S13–S19).

### 3.7. Additional Analysis

As shown in Supporting Information: Tables S4 and S5, meta-regression analyses showed no significant associations between the prevalence of microvascular complications and study-level covariates, including cohort year, mean HbA1c, diabetes duration, insulin dose, age, or lipid levels, in either type 1 or type 2 diabetes (all *p* > 0.05).

## 4. Discussion

This systematic review and meta-analysis quantified the global prevalence of microvascular complications in children and adolescents with type 1 and type 2 diabetes. We found that before age 20, at least one in 10 individuals with diabetes already presents a microvascular complication, with comparable pooled prevalence estimates across diabetes types. This substantial and often under-recognized burden in pediatric diabetes carries implications for both immediate health and long-term risk of severe outcomes [5].

Peripheral nerve abnormalities were common in youth with diabetes, though prevalence varied widely across studies due to differences in diagnostic methods and populations. In type 1 diabetes, pooled prevalence was 22%, consistent with previous reviews reporting 13.5%–62% in youth and young adults [12] and 0%–57.9% in a review combining data from children and adolescents with type 1 and type 2 diabetes [13]. While high HbA1c and longer diabetes duration are known risk factors [12], our meta-regression found no significant associations, suggesting other contributors to early nerve damage and highlighting the need to remain vigilant for complications even in patients who appear well-controlled. In type 2 diabetes, peripheral neuropathy appeared less prevalent, though differences with type 1 diabetes were not statistically significant. The slower manifestation of neuropathy in type 2 diabetes may reflect lower diabetes duration and its multifactorial origins (i.e., hyperglycemia, insulin resistance, obesity, hypertension), compared with the predominantly hyperglycemia-driven nerve damage in type 1 diabetes [76]. A previous meta-analysis found a higher global incidence of diabetes-related lower limb amputations in type 1 diabetes adult patients than in type 2 [77], suggesting that prolonged exposure to the disease may ultimately result in a greater burden of microvascular complications, calling for early action.

Autonomic abnormalities, most often assessed as CAN, were more prevalent in type 1 diabetes, at 32%, yet differences between diabetes types were not statistically significant. This is concerning, as CAN strongly predicts cardiovascular disease and mortality in patients with diabetes [8], but may be reversible with early lifestyle and multifactorial interventions.

Prevalence of diabetic retinopathy was identified in 13.8% of youth with type 1 diabetes and 3% with type 2 diabetes, although not statistically significant. The prevalence in type 2 diabetes is lower than the ~7% reported in a previous meta-analysis [14], likely because that analysis included participants above pediatric age and duplicated cohorts, potentially inflating estimates. The observed differences in prevalence between the two types of diabetes may be attributed to the longer mean diabetes duration in type 1 diabetes (6.2 years) compared with type 2 diabetes (1.5 years), but could also reflect distinct retinal microvascular responses. In type 1 diabetes, early-onset microvascular basement membrane thickening and pericyte loss are prominent, leading to earlier capillary nonperfusion [78]; in type 2 diabetes, retinal changes may be influenced more by concurrent hypertension, dyslipidemia, and low-grade inflammation, which can affect vascular permeability and accelerate macular edema rather than early proliferative changes [79]. Compared with other microvascular complications in youth, retinopathy appears less common, possibly because it typically requires longer cumulative exposure to hyperglycemia and metabolic stress before structural changes become detectable. It could also reflect a lack of screening for retinal disease by clinicians. However, with an estimated global prevalence of about 22% in all ages and rising [80], it is a major cause of adult blindness and vision impairment [81], calling for early screening and proactive management to preserve vision.

Early indicators of renal damage, most often assessed as microalbuminuria, were observed in 13.5% of children and adolescents with type 1 diabetes and 13.8% with type 2 diabetes. A previous review in pediatric type 2 diabetes found a pooled prevalence of albuminuria of 22.17% [15], nearly double our estimate. This discrepancy may reflect their inclusion of duplicate cohort studies, grey literature, and older data from before 2000, which could have overestimated prevalence rates. It may also reflect our broader definition of renal damage, which also included persistent albuminuria and reduced eGFR. Despite being lower than previous estimates, the finding that roughly 1 in 7–8 children or adolescents with any type of diabetes already show early signs of renal damage reveals a substantial clinical burden.

### 4.1. Clinical Implications

Preventing microvascular complications is far more effective than treating them and depends on early identification of individuals at risk. The early years, particularly the transition from prepuberty to puberty when insulin sensitivity declines sharply even in otherwise healthy children [74, 82] represent a critical window for intervention. Maintaining good metabolic stability during this period can prevent or delay endothelial damage and the onset and progression of microvascular complications [83]. Effective prevention strategies should therefore combine education on long-term glycemic and blood pressure management with lifestyle measures, including both structured and unstructured regular physical activity and dietary interventions, to protect endothelial health and optimize metabolic health from the earliest stages of the disease, ensuring proper diabetes care.

## 5. Limitations

There are several limitations to this analysis. First, the included studies varied widely in diagnostic criteria for complications and assessment techniques for retinopathy, neuropathy, and nephropathy. Such methodological variation may contribute to outcome misclassification and residual heterogeneity, and therefore the pooled prevalence estimates should be interpreted with caution. This was also observed in population characteristics, contributing to heterogeneity. Second, smaller sample sizes in the type 2 diabetes group may have reduced statistical power in all studied outcomes, potentially underestimating true prevalence, a limitation that reflects the lower overall number of youths diagnosed with type 2 compared to type 1 diabetes. Likewise, studies often had small sample sizes, which with the lower prevalence of certain complications in this age group, can amplify the effect of publication bias. Third, the limited number of studies reporting key variables restricted the scope of meta-regression. Similarly, important clinical and social determinants (e.g., socioeconomic status, access to care, insulin regimen/adherence, comorbidities) were variably reported and could not be adjusted for, which may have contributed to residual confounding in the pooled estimates. Fourth, subgroup analyses by country income level were limited by the small number of studies for most outcomes. Only peripheral neuropathy in type 1 diabetes allowed a meaningful comparison. For other outcomes, analyses were underpowered, highlighting the need for more geographically balanced evidence. Furthermore, the literature has predominantly reported on the prevalence of CAN, even though urinary, gastrointestinal, and other autonomic dysfunctions are also observed in pediatric and adolescent populations with diabetes [84], and have not been examined. Similarly, neuropathy was assessed either by symptoms or by electrophysiological tests, which can detect nerve damage before symptoms appear and may lead to over or underestimation of prevalence compared with guideline-recommended screening [85]. In addition, other causes of neuropathy and retinal or renal disease unrelated to diabetes could have influenced our prevalence estimates, potentially limiting their accuracy. In addition, the included studies span more than two decades, during which diabetes management and screening practices have evolved substantially. Because few recent studies were available, temporal subgroup analyses were not feasible, and historical differences in care may have influenced prevalence estimates. However, meta-regression using cohort year as a covariate showed no significant association with complication prevalence, suggesting limited temporal effects. Despite these limitations, our review is the first to synthesize global prevalence estimates of microvascular complications specifically in youth with diabetes, with separate analyses for type 1 and type 2 diabetes, including studies of 50 countries.

## 6. Conclusion

Microvascular complications are already present in at least one in 10 young people with diabetes before age 20, with similar prevalence across diabetes types. These findings emphasize the need for earlier and more routine screening in all youth with diabetes, regardless of glycemic stability or disease duration, supporting the implementation of early interventions to prevent or limit further vascular damage.

## Figures and Tables

**Table 1:** Main characteristics of studies including children and adolescents diagnosed with type 1 diabetes.

First author (year)	Country	Sample size (*n* females)	Mean age (years)	Years since diagnosis	Mean % HbA1C	Microvascular complication assessed (events)	Method of assessment	Diagnostic criteria
Abuelwafaa et al. (2019)	Sudan	50 (29)	15	4.92	11.28	Peripheral neuropathy (*n* = 44)	Lower limb NCS	≥2 Abnormal NCS parameters in ≥1 nerve

Amin et al. (2018)	UK	527 (286)	8.8	9.6	9.8	Nephropathy (*n* = 135)	Urinary ACR	ACR 3.5–35 mg/mmol or 4.0–47 mg/mmol in ≥2 samples

Amutha et al. (2019)	India	3252 (614)	15.4	4.2	10.9	Peripheral neuropathy (*n* = 58)	VPT	Mean VPT ≥ 20 V
						Retinopathy (*n* = 240)	Seven-field retinal photography	≥1 Microaneurysm in any field
						Nephropathy (*n* = 353)	ACR	ACR ≥ 30 *μ* g/mg in ≥2 samples

Anderzén et al. (2016)	Sweden	4047	13–18	14.7	4.7	Retinopathy (*n* = 1855)	Fundus photography	Abnormal findings
						Nephropathy (*n* = 290)	Albuminuria	Albuminuria ≥ 20 *μ* g/min

Blankenburg et al. (2012)	Germany	45 (23)	13.2	6.7	8.3	Peripheral neuropathy (*n* = 28)	QST and NCS	Conduction velocity < 41 m/s, amplitude < 5 *μ*V or abnormal QST thresholds

Boysen et al. (2007)	Germany	20 (9)	14	3	8.2	Autonomic neuropathy (*n* = 15)	Cardiorespiratory reflexes	At least 1 abnormal reflex

Briceño et al. (2012)	Venezuela	253 (135)	13.21	5.48	NR	Nephropathy (*n* = 54)	ACR	ACR > 30 mg/g in spot urine

Dabelea et al. (2017)	USA	1746 (867)	17.9	7.9	9.2	Peripheral neuropathy (*n* = 110)	MNSI	MNSI score > 2
						Autonomic neuropathy (*n* = 1615)	HRV	≥3 of 5 abnormalities beyond 5th or 95th percentile vs. age- and sex-matched controls
						Retinopathy (*n* = 71)	Nonmydriatic fundus photography	Abnormal findings
						Nephropathy (*n* = 89)	Albuminuria + eGFR	Creatinine ≥ 30 *μ* g/mg or eGFR ≤ 60 mL/min/1.73 m^2^

Dalla Pozza et al. (2007)	Germany	150 (80)	13.9	—	7.5	Autonomic neuropathy (*n* = 111)	BRS	BRS lower than control group

Damm et al. (2024)	Denmark	105 (48)	15.2	4.8	7.1	Peripheral neuropathy (*n* = 42)	NCS	Threshold stratified by age and height
						Autonomic neuropathy (*n* = 20)	Cardiorespiratory reflexes	At least 1 abnormal reflex

Dart et al. (2014)	Canada	1011 (473)	15.1	NR	9.2	Peripheral neuropathy (*n* = 50)	NR	Abnormal findings
						Autonomic neuropathy (*n* = 568)	Pupillary abnormality	Abnormal findings
						Retinopathy (*n* = 139)	NR	Abnormal findings
						Nephropathy (*n* = 27)	ACR	ACR ≥ 3 mg/mmol

Demirel et al. (2013)	Turkey	155 (88)	14.4	6.3	8.4	Peripheral neuropathy (*n* = 1)	Neurological examination	Abnormal findings
						Retinopathy (*n* = 0)	Fundus examination	Abnormal findings
						Nephropathy (*n* = 25)	Albuminuria (24 h) and renal ultrasound	Albumin > 30 mg/L

Gallardo et al. (2007)	Chile	44 (24)	11.68	3.8	9.1	Nephropathy (*n* = 8)	ACR	ACR > 3.5 mg/mmol (boys) and ACR > 4 mg/mmol (girls)

Ghaemi et al. (2018)	Iran	50 (27)	16.68	8.38	8.6	Peripheral neuropathy (*n* = 12)	Clinical exam + electrodiagnostic studies	Abnormal findings

Gomes et al. (2021)	Brazil	367 (184)	16.4	8.1	9.6	Peripheral neuropathy (*n* = 16)	Clinical exam	Abnormal findings
						Retinopathy (*n* = 28)	Ophtalmoscopy	Abnormal findings
						Nephropathy (*n* = 46)	Albuminuria	Albumin > 30 mg/L

Hajas et al. (2016)	Slovakia	62 (28)	13.9	5.56	8.7	Peripheral neuropathy (*n* = 15)	Clinical symptoms and neurological exam	Abnormal findings

Hasani et al. (2013)	Iran	146 (84)	11.9	3.8	—	Peripheral neuropathy (*n* = 40)	Clinical symptoms and neurological exam	Abnormal findings

Hirschfeld et al. (2015)	Germany	88 (42)	12.7	4.89	8	Peripheral neuropathy (*n* = 43)	NCS	Conduction velocity < 41 m/s, amplitude < 5 *μ*V

Ising et al. (2018)	Sweden	72 (33)	12.8	5.3	7.3	Peripheral neuropathy (*n* = 13)	VPT	≥3 frequencies with z-scores > 1.96 at a site considered pathological

Kamaleldeen et al. (2018)	Egypt	180 (96)	13.82	6	NR	Peripheral neuropathy (*n* = 10)	Neurological examination	Abnormal findings
						Retinopathy (*n* = 2)	Fundus examination (photography or ophthalmoscopy)	Abnormal findings on fundus imaging or ophthalmoscopy
						Nephropathy (*n* = 37)	ACR	Microalbuminuria: 30–300 mg/L

Kardelen et al. (2006)	Turkey	47 (26)	12	4.2	9	Autonomic neuropathy	HRV via holter	HRV lower than control group

Lee et al. (2010)	South Korea	37 (23)	12	NR	9.25	Peripheral neuropathy (*n* = 12)	NCS	Abnormal NCS parameters in ≥2 nerves

Li et al. (2016)	USA	25,218	<18	NR	NR	Nephropathy (*n* = 868)	Clinical codes	Clinical codes

Louraki et al. (2016)	Greece	85 (40)	13.5	5.5	8.2	Peripheral neuropathy (*n* = 29)	NCS	Abnormal findings

Machado et al. (2013)	Uruguay	192	<15	5.8	NR	Nephropathy (*n* = 10)	Microalbuminuria	Albumin > 30 mg/L
						Retinopathy (*n* = 1)	Fundus examination	Abnormal findings

Mandilou et al. (2020)	Congo	62 (40)	16.84	5.12	10.8	Peripheral neuropathy (*n* = 1)	MNSI	MNSI score > 2.5
						Retinopathy (*n* = 4)	Fundus examination	Abnormal findings
						Nephropathy (*n* = 13)	ACR	ACR > 30 mg/g

Marshall et al. (2012)	Rwanda	286	18.6	3.4	11.2	Peripheral neuropathy (*n* = 5)	Clinical exam	Abnormal findings
						Nephropathy (*n* = 31)	ACR	ACR > 30 mg/g

Metwalley et al. (2018)	Egypt	60 (39)	15.1	7.95	9.7	Autonomic neuropathy (*n* = 22)	Cardiorespiratory reflexes	At least 1 abnormal reflex

Msanga et al. (2020)	Tanzania	155 (77)	17	NR	NR	Peripheral neuropathy (*n* = 8)	Clinical exam	Abnormal findings
						Autonomic neuropathy (*n* = 16)	Cardiorespiratory reflexes	At least 1 abnormal reflex
						Retinopathy (*n* = 16)	Fundus examination	eGFR < 60 mL/min/1.73 m^2^
						Nephropathy (*n* = 51)	Proteinuria	Abnormal findings

Najem et al. (2021)	Tanzania	148 (82)	8.9	3.6	10.6	Peripheral neuropathy (*n* = 35)	Clinical exam	Abnormal findings

Nelson et al. (2006)	Canada	73 (35)	13.7	8.1	9	Peripheral neuropathy (*n* = 42)	NCS	<2 Standard deviations below the mean in ≥2 nerves

Nordwall et al. (2006)	Sweden	80 (33)	18.7	11.6	7.3	Peripheral neuropathy (*n* = 47)	NCS	Abnormal findings in > 2 nerves
						Retinopathy (*n* = 22)	Fundus examination	Abnormal findings
						Nephropathy (*n* = 4)	AER	AER > 20 *μ* g/min

Ogugua et al. (2019)	Nigeria	18 (NR)	14.2	1.98	NR	Retinopathy (*n* = 4)	Mydriatic ophthalmoscopy	Abnormal findings
						Nephropathy (*n* = 8)	ACR	ACR of 2.5–25 mg/mmol or 30–300 *μ* g/mL (spot urine) in males and 3.5–25 mg/mmol in females

Ou et al. (2017)	Taiwan	1486 (772)	7.69	NR	NR	Peripheral neuropathy (*n* = 87)	Clinical codes	Clinical codes
						Retinopathy (*n* = 663)		
						Nephropathy (*n* = 195)		

Piccoli et al. (2025)	Italy	81 (35)	15.7	9.2	7.2	Peripheral neuropathy (*n* = 11)	MNSI	Age-dependent cut-off values
						Autonomic neuropathy (*n* = 7)	Cardiorespiratory reflexes	

Rasmussen et al. (2023)	Denmark	60 (30)	16.9	8.5	7.6	Peripheral neuropathy (*n* = 11)	Clinical exam	Abnormal findings
						Autonomic neuropathy (*n* = 7)	QSART, Cardiorespiratory reflexes, and tilt Table test analyzing orthostatic parameters	Abnormal findings

Sandhu et al. (2020) 2003 cohort	New Zealand	237 (128)	16.7	7.2	7.34	Peripheral neuropathy (*n* = 7)	NR	Abnormal findings
						Retinopathy (*n* = 58)	Fundus examination	Abnormal findings
						Nephropathy (*n* = 60)	ACR	ACR > 30 mg/mmol

Sandhu et al. (2020) 2017 cohort	New Zealand	255 (107)	15.6	6.1	7.1	Peripheral neuropathy (*n* = 8)	NR	Abnormal findings
						Retinopathy (*n* = 15)	Fundus examination	Abnormal findings
						Nephropathy (*n* = 15)	ACR	ACR > 30 mg/mmol

Singh et al. (2021)	India	50 (24)	12.2	5.1	9.14	Peripheral neuropathy (*n* = 28)	NCS	Abnormal if the patient could not sense a 10-g monofilament

Singh et al. (2022)	India	66 (22)	11.1	4	8.7	Peripheral neuropathy (*n* = 60)	NCS	Abnormal findings in > 2 nerves

Son et al. (2015)	South Korea	109 (70)	17.5	9.5	8.3	Nephropathy (*n* = 23)	Spot urine ACR	ACR > 30–300 mg/g

Toopchizadeh et al. (2016)	Iran	40 (25)	12.73	6.63	8.46	Peripheral neuropathy (*n* = 23)	NCS	If values were beyond mean ± 2.5 standard deviation of controls

Unnikrishnan et al. (2008)	India	535 (251)	17.6	5.6	9.3	Peripheral neuropathy (*n* = 32)	Clinical exam	24-h urine protein excretion of > 500 mg per 24 h or the presence of microalbuminuria
						Retinopathy (*n* = 27)	Ophtalmoscopy	At least 1 abnormal finding
						Nephropathy (*n* = 29)	Protein excretion	≥2 HRV abnormalities (based on ≤5th or ≥95th percentiles of age- and sex-matched local control subjects)

Varley et al. (2022)	Australia	1153 (1136)	16.5	8	8.7	Peripheral neuropathy (*n* = 281)	Thermal threshold testing	≥1 Microaneurysm in any field
						Autonomic neuropathy (*n* = 309)	HRV	ACR ≥ 1.0 mg/mmol (male) and ≥1.4 mg/mmol (female)
						Retinopathy (*n* = 173)	7-Field fundus photography	At least 1 abnormal finding
						Nephropathy (*n* = 33)	ACR	≥2 HRV abnormalities (based on ≤5th or ≥95th percentiles of age- and sex-matched local control subjects)

Verkauskiene et al. (2016)	Lithuania	1209 (623)	15	5.1	8.7	Peripheral neuropathy (*n* = 100)	MNSI and clinical exam	≥2 abnormal findings
						Retinopathy (*n* = 96)	Fundus photography	Abnormal findings
						Nephropathy (*n* = 95)	AER	AER > 30–300 mg/24 h

Walter-Höliner et al. (2018)	Austria	38 (17)	12.6	5.6	8.1	Peripheral neuropathy (*n* = 12)	NCS	Conduction velocity < 42 m/s in the peroneal and medial plantar nerve, <40 m/s in the tibial motor nerve, less than 49 m/s in the median motor, and <44 m/s in the median sensory nerve

Winter et al. (2024)	New Zealand	646 (302)	12.6	5.2	8.6	Retinopathy (*n* = 359)	Fundus photography	Abnormal findings

Abbreviations: ACR, albumin–creatinine ratio; AER, albumin excretion ratio; BRS, baroreflex sensitivity; eGFR, estimated glomerular filtration rate; HRV, heart rate variability; MNSI, Michigan Neuropathy Screening Instrument; NCS, nerve conduction studies; NR, not reported; QSART, quantitative sudomotor axon reflex test; QST, quantitative sensory testing; VPT, vibration perception threshold.

**Table 2:** Main characteristics of studies including children and adolescents diagnosed with type 2 diabetes.

First author (year)	Country	Sample size (*n* females)	Mean age (years)	Years since diagnosis	Mean %HbA1C	Microvascular complication assessed (events)	Method of assessment	Prevalence
Amed et al. (2012)	Canada	221 (130)	13.9	NR	9.5	Nephropathy (*n* = 32)	Microalbuminuria	Microalbuminuria

Chuback et al. (2006)	Canada	110 (71)	15	2.5	8	Peripheral neuropathy (*n* = 0)	Clinical exam	Abnormal finding

Copeland et al. (2011)	USA	704 (454)	14	0.65	5.9	Nephropathy (*n* = 92)	ACR	ACR > 30 mg/g

Dart et al. (2014)	Canada	342 (213)	16.5	NR	8.9	Peripheral neuropathy (*n* = 342)	NR	Abnormal findings
						Autonomic neuropathy (*n* = 13)	Pupillary abnormality	Abnormal findings
						Retinopathy (*n* = 40)	NR	Abnormal findings
						Nephropathy (*n* = 30)	ACR	ACR ≥ 3 mg/mmol

Ettinger et al. (2005)	USA	26 (14)	15	1.5	7.4	Nephropathy (*n* = 10)	ACR	Albumin > 30 mg/day

Li et al. (2016)	USA	5324 (3194)	<18	NR	NR	Nephropathy (*n* = 184)	Clinical codes	Clinical codes

Nambam et al. (2017)	USA	598 (376)	16	NR	7.6	Nephropathy (*n* = 36)	Microalbuminuria	Microalbuminuria

Osman et al. (2013)	Sudan	38 (20)	NR	NR	9.1	Peripheral neuropathy (*n* = 38)	Clinical reports	Clinical reports
						Retinopathy (*n* = 0)		
						Nephropathy (*n* = 7)		

Ruhayel et al. (2010)	Australia	33 (22)	13.4	NR	7	Retinopathy (*n* = 4)	Clinical reports	ACR ≥ 3.5 mg/mmol creatinine
						Nephropathy (*n* = 9)	ACR	

Shah et al. (2009)	USA	397 (257)	<20	7.7	8.8	Autonomic neuropathy (*n* = 32)	HRV	≥3 (out of five) HRV indices were greater or less than 2.5 SDs compared with control adolescents

Shield et al. (2009)	UK	67 (40)	14.5	NR	7.5	Retinopathy (*n* = 0)	Clinical records	Clinical records
						Nephropathy (*n* = 2)		

Son et al. (2015)	South Korea	18 (14)	15.4	0.9	8.8	Nephropathy (*n* = 8)	Spot urine ACR	ACR > 30–300 mg/g

Thanh et al. (2015)	USA	86 (59)	14.2	NR	7	Nephropathy (*n* = 14)	Urine albumin	> 30 *μ* g/mg of creatinine

TODAY study group (2021)	USA	674	14	<2	6.1	Peripheral neuropathy (*n* = 674)	MNSI	MNSI ≥ 3

Unnikrishnan et al. (2008)	India	36 (15)	18.9	2.7	8.7	Peripheral neuropathy (*n* = 36)	Clinical exam	Abnormal findings
						Retinopathy (*n* = 0)	Ophtalmoscopy	Abnormal findings
						Nephropathy (*n* = 0)	Protein excretion	24-h urine protein excretion of > 500 mg/24 h or the presence of microalbuminuria

Varley et al. (2022)	Australia	66 (41)	15.4	1.7	7.1	Peripheral neuropathy (*n* = 66)	Thermal threshold testing	At least 1 abnormal finding
						Autonomic neuropathy (*n* = 31)	HRV	≥2 HRV abnormalities (based on ≤5th or ≥95th percentiles of age- and sex-matched local control subjects)
						Retinopathy (*n* = 1)	7-field fundus photography	≥1 Microaneurysm in any field
						Nephropathy (*n* = 10)	ACR	ACR ≥ 1.0 mg/mmol (male) and ≥1.4 mg/mmol (female)

Abbreviations: ACR, albumin–creatinine ratio; CAN, cardiac autonomic neuropathy; HRV, heart rate variability; MNSI, Michigan Neuropathy Screening Instrument; NR, not reported.

**Table 3:** Prevalence estimates of microvascular complications in children and adolescents with type 1 and type 2 diabetes.

Microvascular complication	Prevalence (95% CI)		Between-group *p*-value
	Type 1 diabetes	Type 2 diabetes	
Peripheral neuropathy	22.02% (16.86–27.75)	11.04% (2.73–23.45)	0.104
Autonomic neuropathy	31.98% (11.13–57.44)	15.37% (3.09–34.29)	0.252
Retinopathy	13.76% (6.43–23.24)	2.97% (0.00–10.33)	0.057
Nephropathy	13.70% (10.25–17.54)	12.63% (7.99–18.07)	0.845

## Data Availability

The data that support the findings of this review are available upon reasonable request from the corresponding author (Yasmin Ezzatvar).

## Supporting Information

Supporting Information Table S1: Literature search strategy for all the databases. Table S2: Excluded studies and reasons for exclusion. Table S3: Results of the Joanna Briggs Institute Appraisal Checklist for Cohort studies. Figure S1: Forest plot showing separate prevalence estimates of peripheral neuropathy in children and adolescents with type 1 diabetes and those with type 2 diabetes. Figure S2: Forest plot showing separate prevalence estimates of autonomic neuropathy in children and adolescents with type 1 diabetes and those with type 2 diabetes. Figure S3: Forest plot showing separate prevalence estimates of retinopathy in children and adolescents with type 1 diabetes and those with type 2 diabetes. Figure S4: Forest plot showing separate prevalence estimates of nephropathy in children and adolescents with type 1 diabetes and those with type 2 diabetes. Table S4: Results of random-effects meta-regressions for the prevalence of microvascular complications in children and adolescents with type 1 diabetes. Table S5: Results of random-effects meta-regressions for the prevalence of microvascular complications in children and adolescents with type 2 diabetes. Figures S5–S12: Leave-one-out sensitivity analyses. Figures S13–S19: Publication bias analyses.
