# Supplementary material for: Global Prevalence of Microvascular Complications in Children and Adolescents With Type 1 and Type 2 Diabetes: A Systematic Review and Meta-Analysis
Source: Pediatr Diabetes. 2025 Nov 26;2025:8398194. doi: 10.1155/pedi/8398194 (PMC12674870; doi:10.1155/pedi/8398194)
Supplement: Supporting Information — Table S1: Literature search strategy for all the databases. Table S2: Excluded studies and reasons for exclusion. Table S3: Results of the Joanna Briggs Institute Appraisal Checklist for Cohort studies. Figure S1: Forest plot showing separate prevalence estimates of peripheral neuropathy in children and adolescents with type 1 diabetes and those with type 2 diabetes. Figure S2: Forest plot showing separate prevalence estimates of autonomic neuropathy in children and adolescents with type 1 diabetes and those with type 2 diabetes. Figure S3: Forest plot showing separate prevalence estimates of retinopathy in children and adolescents with type 1 diabetes and those with type 2 diabetes. Figure S4: Forest plot showing separate prevalence estimates of nephropathy in children and adolescents with type 1 diabetes and those with type 2 diabetes. Table S4: Results of random-effects meta-regressions for the prevalence of microvascular complications in children and adolescents with type 1 diabetes. Table S5: Results of random-effects meta-regressions for the prevalence of microvascular complications in children and adolescents with type 2 diabetes. Figures S5–S12: Leave-one-out sensitivity analyses. Figures S13–S19: Publication bias analyses. [file 8398194.f1.docx]

**Global Prevalence of Microvascular Complications in Children and Adolescents with Type 1 and Type 2 Diabetes: A Systematic Review and Meta-Analysis.**

Contents

[Table S1. Literature search strategy for all the databases 3](#_Toc212544995)

[Table S2. Excluded studies and reasons for exclusion. 4](#_Toc212544996)

[Table S3. Results of the Joanna Briggs Institute Appraisal Checklist for Cohort studies. 9](#_Toc212544997)

[Figure S1. Forest plot showing separate prevalence estimates of peripheral neuropathy in children and adolescents with type 1 diabetes and those with type 2 diabetes. 11](#_Toc212544998)

[Figure S2. Forest plot showing separate prevalence estimates of autonomic neuropathy in children and adolescents with type 1 diabetes and those with type 2 diabetes. 12](#_Toc212544999)

[Figure S3. Forest plot showing separate prevalence estimates of retinopathy in children and adolescents with type 1 diabetes and those with type 2 diabetes. 13](#_Toc212545000)

[Figure S4. Forest plot showing separate prevalence estimates of nephropathy in children and adolescents with type 1 diabetes and those with type 2 diabetes. 14](#_Toc212545001)

[Figure S5. Forest plot showing separate prevalence estimates of peripheral neuropathy in children and adolescents with type 1 diabetes according to country income. 15](#_Toc212545002)

[Table S4. Results of random-effects meta-regressions for the prevalence of microvascular complications in children and adolescents with type 1 diabetes. 16](#_Toc212545003)

[Table S5. Results of random-effects meta-regressions for the prevalence of microvascular complications in children and adolescents with type 2 diabetes. 17](#_Toc212545004)

[Figure S5. Leave-one-out sensitivity analysis for prevalence of peripheral neuropathy in type 1 diabetes. 18](#_Toc212545005)

[Figure S6. Leave-one-out sensitivity analysis for prevalence of autonomic neuropathy in type 1 diabetes. 19](#_Toc212545006)

[Figure S7. Leave-one-out sensitivity analysis for prevalence of retinopathy in type 1 diabetes. 20](#_Toc212545007)

[Figure S8. Leave-one-out sensitivity analysis for prevalence of nephropathy in type 1 diabetes. 21](#_Toc212545008)

[Figure S9. Leave-one-out sensitivity analysis for prevalence of peripheral neuropathy in type 2 diabetes. 22](#_Toc212545009)

[Figure S10. Leave-one-out sensitivity analysis for prevalence of autonomic neuropathy in type 2 diabetes. 23](#_Toc212545010)

[Figure S11. Leave-one-out sensitivity analysis for prevalence of retinopathy in type 2 diabetes. 24](#_Toc212545011)

[Figure S12. Leave-one-out sensitivity analysis for prevalence of nephropathy in type 2 diabetes. 25](#_Toc212545012)

[Figure S13. Doi plots for peripheral neuropathy in type 1 diabetes patients. 26](#_Toc212545013)

[Figure S14. Doi plots for peripheral neuropathy in type 2 diabetes patients. 27](#_Toc212545014)

[Figure S15. Doi plots for autonomic neuropathy in type 1 diabetes patients. 28](#_Toc212545015)

[Figure S16. Doi plots for retinopathy in type 1 diabetes patients. 29](#_Toc212545016)

[Figure S17. Doi plots for retinopathy in type 2 diabetes patients. 30](#_Toc212545017)

[Figure S18. Doi plots for nephropathy in type 1 diabetes patients. 31](#_Toc212545018)

[Figure S19. Doi plots for nephropathy in type 2 diabetes patients. 32](#_Toc212545019)

This supplemental material has been provided by the authors to give readers additional information about their work.

# **Table S1.** Literature search strategy for all the databases

| PubMed:  (“type 1 diabetes” OR “type 2 diabetes”) AND (“children” OR “adolescents” OR “youth”) AND (“neuropathy” OR “peripheral neuropathy” OR “autonomic neuropathy” OR “diabetic complications” OR “microvascular complications” OR “retinopathy” OR “nephropathy”) AND (“prevalence” OR “frequency” OR “epidemiology”)  From 2000 to 2025 | 738 results |
| --- | --- |
| Embase:  ('type 1 diabetes mellitus' OR 'type 2 diabetes mellitus' OR 'type 1 diabetes' OR 'type 2 diabetes') AND ('neuropathy' OR 'peripheral neuropathy' OR 'autonomic neuropathy' OR 'diabetic complication' OR 'microvascular complication' OR 'retinopathy' OR 'nephropathy') AND ([adolescent]/lim OR [child]/lim OR [infant]/lim OR [newborn]/lim OR [preschool]/lim OR [school]/lim) AND [2000-2025]/py AND ('cohort analysis'/de OR 'cross sectional study'/de OR 'observational study'/de OR 'prospective study'/de OR 'retrospective study'/de) | 1,186 results |
| Web of Science:  Topic = ("type 1 diabetes" OR "type 2 diabetes")  AND Topic = (children OR adolescents OR youth OR teen* OR pediatric)  AND Topic = ("neuropathy" OR "peripheral neuropathy" OR "autonomic neuropathy" OR "diabetic complications" OR "microvascular" OR "retinopathy" OR "nephropathy")  Timespan: 2000-01-01 to 2025-08-10 (Index Date) | 1,694 results |

# **Table S2.** Excluded studies and reasons for exclusion.

| **Reason** | **Explanation** | **Refs.** |
| --- | --- | --- |
| Cohorts with data prior to 2000 | Studies conducted before the year 2000 were excluded because treatment approaches for diabetes in children and adolescents have changed substantially since then. Earlier studies may not reflect current standards of care, which limits their comparability with more recent research. | 1-17 |
| Cohorts including participants >20 years old | Studies that reported data on broader age ranges, including young adults, were excluded, since disease characteristics, treatment approaches, and complication risks in children and adolescents, differ from those of adults. | 18-21 |
| Cohorts combining individuals with type 1 and type 2 diabetes | Cohorts combining individuals with type 1 and type 2 diabetes were excluded, as the two types differ in pathophysiology, age at onset, treatment strategies, risk of complications, and disease progression, which could confound analyses if studied together. | 22, 23 |
| Duplicated cohorts | **The Children's Hospital at Westmead:**  To avoid overlapping data from studies conducted at the same center (the Children’s Hospital at Westmead, Sydney, Australia), only the most recent and comprehensive study (Varley et al., 2022) was included. Although previous studies (e.g., Craig et al., Cho et al., Aulich et al., Eppens et al. Mohsin et al., Virk et al.) are foundational, they draw from the same cohort. The Varley study was selected because it includes a larger sample size, a longer follow-up period, and more detailed reporting of outcomes relevant to this review. | 24-29 |
|  | **SEARCH study:**  To avoid overlapping data from the SEARCH for Diabetes in Youth study, only the most complete publication (Dabelea et al., 2017) was included. Although previous studies (e.g., Bell et al., Maahs et al., Mayer-Davis et al., Jaiswal et al., Sauder et al.) provide important insights, they draw from the same registry and cohort. The study by Dabelea et al. was selected because it includes a larger sample, longer follow-up, and a broader assessment of diabetes complications, making it the most relevant to this review. | 30-35 |
|  | **DER-CA cohort:**  To avoid overlapping data from the Diabetes Education Resource for Children and Adolescents (DER-CA) cohort in Manitoba, only the most comprehensive study (Dart et al., 2014) was included. Although Sellers et al. (2009) reported important findings, it was based on a smaller retrospective case series from the same clinic population. Dart et al. (2014) was selected because it leveraged the full DER-CA registry, linked with provincial health records, and provided a broader assessment of complications, making it more relevant to this review. | 36 |

**References:**

1. Bognetti, E., Calori, G., Meschi, F., Macellaro, P., Bonfanti, R., & Chiumello, G. (1997). Prevalence and correlations of early microvascular complications in young type I diabetic patients: role of puberty. Journal of Pediatric Endocrinology and Metabolism, 10(6), 587-592.
2. Davis, E. A., Jones, T. W., Walsh, P., & Byrne, G. C. (1997). The use of biothesiometry to detect neuropathy in children and adolescents with IDDM. Diabetes care, 20(9), 1448-1453.
3. Donaghue, K. C., Fung, A. T. W., Fairchild, J. M., Howard, N. J., & Silink, M. (1996). Prospective assessment of autonomic and peripheral nerve function in adolescents with diabetes. Diabetic medicine, 13(1), 65-71.
4. Hotu, S., Carter, B., Watson, P. D., Cutfield, W. S., & Cundy, T. (2004). Increasing prevalence of type 2 diabetes in adolescents. Journal of paediatrics and child health, 40(4), 201-204.
5. Hyllienmark, L., Brismar, T., & Ludvigsson, J. (1995). Subclinical nerve dysfunction in children and adolescents with IDDM. Diabetologia, 38(6), 685-692.
6. Jefferies, C., Carter, P., Reed, P. W., Cutfield, W., Mouat, F., Hofman, P. L., & Gunn, A. J. (2012). The incidence, clinical features, and treatment of type 2 diabetes in children< 15 yr in a population‐based cohort from Auckland, New Zealand, 1995–2007. Pediatric diabetes, 13(4), 294-300.
7. Käär, M. L., Saukkonen, A. L., Pitkänen, M., & Åkerblom, H. K. (1983). PERIPHERAL NEUROPATHY IN DIABETIC CHILDREN AND ADOLESCENTS: A Cross‐sectional Study. Acta Paediatrica, 72(3), 373-378.
8. Karavanak, K., & Baum, J. D. (1999). Prevalence of microvascular and neurologic abnormalities in a population of diabetic children. Journal of Pediatric Endocrinology and Metabolism, 12(3), 411-422.
9. Kim, N. H., Pavkov, M. E., Knowler, W. C., Hanson, R. L., Weil, E. J., Curtis, J. M., ... & Nelson, R. G. (2010). Predictive value of albuminuria in American Indian youth with or without type 2 diabetes. Pediatrics, 125(4), e844-e851.
10. Maser, R. E., Steenkiste, A. R., Dorman, J. S., Nielsen, V. K., Bass, E. B., Manjoo, Q., ... & Orchard, T. J. (1989). Epidemiological correlates of diabetic neuropathy: report from Pittsburgh Epidemiology of Diabetes Complications Study. Diabetes, 38(11), 1456-1461.
11. Massin, M. M., Derkenne, B., Tallsund, M., Rocour-Brumioul, D., Ernould, C., Lebrethon, M. C., & Bourguignon, J. P. (1999). Cardiac autonomic dysfunction in diabetic children. Diabetes Care, 22(11), 1845-1850.
12. Olsen, B. S., Johannesen, J., Sjølie, A. K., Borch‐Johnsen, K., Hougaard, P., Thorsteinsson, B., ... & Childhood, I. (1999). Metabolic control and prevalence of microvascular complications in young Danish patients with Type 1 diabetes mellitus. Diabetic Medicine, 16(1), 79-85.
13. Ringel, R. E., Chalew, S. A., Armour, K. A., McLughun, J., McCarter jr, R. J., & Kramer, W. E. (1993). Cardiovascular reflex abnormalities in children and adolescents with diabetes mellitus. Diabetes Care, 16(5), 734-741.
14. Solders, G., Thalme, B., Aguirre‐Aquino, M., Brandt, L., Berg, U., & Persson, A. (1997). Nerve conduction and autonomic nerve function in diabetic children. A 10‐year follow‐up study. Acta Paediatrica, 86(4), 361-366.
15. Tesfaye, S., Stevens, L. K., Stephenson, J. M., Fuller, J. H., Plater, M., Ionescu-Tirgoviste, C., ... & EURODIAB IDDM Complications Study Group. (1996). Prevalence of diabetic peripheral neuropathy and its relation to glycaemic control and potential risk factors: the EURODIAB IDDM Complications Study. Diabetologia, 39(11), 1377-1384.
16. Verrotti, A., Chiarelli, F., Blasetti, A., & Morgese, G. (1995). Autonomic neuropathy in diabetic children. Journal of paediatrics and child health, 31(6), 545-548.
17. Young, R. J., Macintyre, C. C. A., Martyn, C. N., Prescott, R. J., Ewing, D. J., Smith, A. F., ... & Clarke, B. F. (1986). Progression of subclinical polyneuropathy in young patients with type 1 (insulin-dependent) diabetes: associations with glycaemic control and microangiopathy (microvascular complications). Diabetologia, 29(3), 156-161.
18. Christensen, M. M. B., Hommel, E. E., Jørgensen, M. E., Von Scholten, B. J., Fleischer, J., & Hansen, C. S. (2018). Prevalence of diabetic neuropathy in young adults with type 1 diabetes and the association with insulin pump therapy. Diabetes technology & therapeutics, 20(12), 787-796.
19. Dart, A. B., Wicklow, B., Blydt-Hansen, T. D., Sellers, E. A., Malik, S., Chateau, D., ... & McGavock, J. M. (2019). A holistic approach to risk for early kidney injury in indigenous youth with type 2 diabetes: a proof of concept paper from the iCARE cohort. Canadian Journal of Kidney Health and Disease, 6, 2054358119838836.
20. Farah, S. E., Wals, K. T., Friedman, I. B., Pisacano, M. A., & DiMartino-Nardi, J. (2006). Prevalence of retinopathy and microalbuminuria in pediatric type 2 diabetes mellitus. Journal of Pediatric Endocrinology and Metabolism, 19(7), 937-942.
21. Yeow, T. P., Aun, E. S. Y., Hor, C. P., Lim, S. L., Khaw, C. H., & Aziz, N. A. (2019). Challenges in the classification and management of Asian youth-onset diabetes mellitus-lessons learned from a single centre study. PloS one, 14(1), e0211210.
22. El Samahy, M. H., Elbarbary, N. S., & Elmorsi, H. M. (2015). Current status of diabetes management, glycemic control and complications in children and adolescents with diabetes in Egypt. Where do we stand now? And where do we go from here?. Diabetes Research and Clinical Practice, 107(3), 370-376.
23. Zabeen, B., Nahar, J., Islam, N., Azad, K., & Donaghue, K. (2018). Risk factors associated with microalbuminuria in children and adolescents with diabetes in Bangladesh. Indian journal of endocrinology and metabolism, 22(1), 85-88.
24. Aulich, J., Cho, Y. H., Januszewski, A. S., Craig, M. E., Selvadurai, H., Wiegand, S., ... & Donaghue, K. C. (2019). Associations between circulating inflammatory markers, diabetes type and complications in youth. Pediatric Diabetes, 20(8), 1118-1127
25. Cho, Y. H., Craig, M. E., Hing, S., Gallego, P. H., Poon, M., Chan, A., & Donaghue, K. C. (2011). Microvascular complications assessment in adolescents with 2‐to 5‐yr duration of type 1 diabetes from 1990 to 2006. Pediatric diabetes, 12(8), 682-689.
26. Craig, M. E., Duffin, A. C., Gallego, P. H., Lam, A., Cusumano, J., Hing, S., & Donaghue, K. C. (2008). Plantar fascia thickness, a measure of tissue glycation, predicts the development of complications in adolescents with type 1 diabetes. Diabetes care, 31(6), 1201-1206.
27. Eppens, M. C., Craig, M. E., Cusumano, J., Hing, S., Chan, A. K., Howard, N. J., ... & Donaghue, K. C. (2006). Prevalence of diabetes complications in adolescents with type 2 compared with type 1 diabetes. Diabetes care, 29(6), 1300-1306.
28. Mohsin, F., Craig, M. E., Cusumano, J., Chan, A. K., Hing, S., Lee, J. W., ... & Donaghue, K. C. (2005). Discordant trends in microvascular complications in adolescents with type 1 diabetes from 1990 to 2002. Diabetes care, 28(8), 1974-1980.
29. Virk, S. A., Donaghue, K. C., Cho, Y. H., Benitez-Aguirre, P., Hing, S., Pryke, A., ... & Craig, M. E. (2016). Association between HbA1c variability and risk of microvascular complications in adolescents with type 1 diabetes. The Journal of Clinical Endocrinology & Metabolism, 101(9), 3257-3263.
30. Bell, R. A., Mayer-Davis, E. J., Beyer, J. W., D'Agostino Jr, R. B., Lawrence, J. M., Linder, B., ... & SEARCH for Diabetes in Youth Study Group. (2009). Diabetes in non-Hispanic white youth: prevalence, incidence, and clinical characteristics: the SEARCH for Diabetes in Youth Study. Diabetes care, 32(Supplement_2), S102-S111.
31. Jaiswal, M., Divers, J., Dabelea, D., Isom, S., Bell, R. A., Martin, C. L., ... & Feldman, E. L. (2017). Prevalence of and risk factors for diabetic peripheral neuropathy in youth with type 1 and type 2 diabetes: SEARCH for diabetes in youth study. Diabetes care, 40(9), 1226-1232.
32. Jaiswal, M., Divers, J., Urbina, E. M., Dabelea, D., Bell, R. A., Pettitt, D. J., ... & SEARCH for Diabetes in Youth Study Group. (2018). Cardiovascular autonomic neuropathy in adolescents and young adults with type 1 and type 2 diabetes: The SEARCH for Diabetes in Youth Cohort Study. Pediatric diabetes, 19(4), 680-689.
33. Maahs, D. M., Snively, B. M., Bell, R. A., Dolan, L., Hirsch, I., Imperatore, G., ... & Dabelea, D. (2007). Higher prevalence of elevated albumin excretion in youth with type 2 than type 1 diabetes: the SEARCH for Diabetes in Youth study. Diabetes care, 30(10), 2593-2598.
34. Mayer-Davis, E. J., Beyer, J., Bell, R. A., Dabelea, D., D'Agostino Jr, R., Imperatore, G., ... & SEARCH for Diabetes in Youth Study Group. (2009). Diabetes in African American youth: prevalence, incidence, and clinical characteristics: the SEARCH for Diabetes in Youth Study. Diabetes care, 32(Supplement_2), S112-S122.
35. Sauder, K. A., Stafford, J. M., Mayer-Davis, E. J., Jensen, E. T., Saydah, S., Mottl, A., ... & Yi-Frazier, J. (2019). Co-occurrence of early diabetes-related complications in adolescents and young adults with type 1 diabetes: an observational cohort study. The Lancet Child & Adolescent Health, 3(1), 35-43.
36. Sellers, E. A., Blydt-Hansen, T. D., Dean, H. J., Gibson, I. W., Birk, P. E., & Ogborn, M. (2009). Macroalbuminuria and renal pathology in First Nation youth with type 2 diabetes. Diabetes care, 32(5), 786-790.

# **Table S3.** Results of the Joanna Briggs Institute Appraisal Checklist for Cohort studies.

| **JBI Appraisal Checklist for Prevalence studies** | | | | | | | | | | |
| --- | --- | --- | --- | --- | --- | --- | --- | --- | --- | --- |
| Study | 1 | 2 | 3 | 4 | 5 | 6 | 7 | 8 | 9 | Total Score |
| Abuelwafaa et al. (2019) | Y | N | N | Y | Y | Y | Y | Y | Y | 7/9 |
| Amed et al. (2012) | Y | Y | Y | Y | Y | Y | Y | Y | Y | 9/9 |
| Amin et al. (2018) | Y | N | Y | Y | Y | Y | Y | Y | Y | 8/9 |
| Amutha et al. (2019) | Y | N | Y | Y | Y | Y | Y | Y | Y | 8/9 |
| Anderzén et al. (2016) | Y | Y | Y | Y | Y | Y | Y | Y | Y | 9/9 |
| Blankenburg et al. (2012) | Y | N | N | Y | Y | Y | Y | Y | NR | 6/9 |
| Boysen et al. (2007) | Y | N | N | Y | Y | Y | Y | Y | NR | 6/9 |
| Chuback et al. (2006) | Y | N | N | Y | Y | Y | Y | Y | Y | 7/9 |
| Copeland et al. (2011) | Y | Y | Y | Y | Y | Y | Y | Y | Y | 9/9 |
| Dabelea et al. (2017) | Y | Y | Y | Y | Y | Y | Y | Y | Y | 9/9 |
| Dalla Pozza et al. (2007) | Y | N | Y | Y | Y | Y | Y | Y | Y | 8/9 |
| Damm et al. (2024) | Y | N | N | Y | Y | Y | Y | Y | Y | 7/9 |
| Dart et al. (2014) | Y | Y | Y | Y | Y | Y | Y | Y | NR | 8/9 |
| Demirel et al. (2013) | Y | N | Y | Y | Y | Y | Y | Y | NR | 7/9 |
| Ettinger et al. (2005) | Y | N | N | Y | Y | Y | Y | Y | Y | 7/9 |
| Gallardo et al. (2007) | Y | N | N | Y | Y | Y | Y | Y | Y | 7/9 |
| Ghaemi et al. (2018) | Y | N | N | Y | Y | Y | Y | Y | NR | 6/9 |
| Gomes et al. (2021) | Y | N | Y | Y | Y | Y | Y | Y | Y | 8/7 |
| Hajas et al. (2016) | Y | N | N | Y | Y | Y | Y | Y | NR | 6/9 |
| Hasani et al. (2013) | Y | N | Y | Y | Y | Y | Y | Y | NR | 8/9 |
| Hirschfeld et al. (2015) | Y | N | N | Y | Y | Y | N | Y | NR | 6/9 |
| Ising et al. (2018) | Y | N | N | Y | Y | Y | Y | Y | NR | 6/9 |
| Kamaleldeen et al. (2018) | Y | N | Y | Y | Y | Y | Y | Y | NR | 7/9 |
| Kardelen et al. (2006) | Y | N | N | Y | Y | Y | Y | Y | Y | 7/9 |
| Le et al. (2015) | Y | N | N | Y | Y | Y | Y | Y | Y | 7/9 |
| Lee et al. (2010) | Y | N | N | Y | Y | Y | Y | Y | Y | 7/9 |
| Li et al. (2016) | Y | N | Y | Y | Y | Y | Y | Y | Y | 8/9 |
| Louraki et al. (2016) | Y | N | N | Y | Y | Y | Y | Y | Y | 7/9 |
| Machado et al. (2013) | Y | N | Y | Y | Y | Y | Y | Y | Y | 8/9 |
| Mandilou et al. (2020) | Y | N | N | Y | Y | Y | Y | Y | NR | 6/9 |
| Marshall et al. (2012) | Y | N | Y | Y | Y | Y | Y | Y | NR | 7/9 |
| Metwalley et al. (2018) | Y | N | N | Y | Y | Y | Y | Y | NR | 6/9 |
| Msanga et al. (2020) | Y | N | Y | Y | Y | Y | Y | Y | Y | 8/9 |
| Najem et al. (2021) | Y | N | Y | Y | Y | Y | Y | Y | Y | 8/9 |
| Nambam et al. (2017) | Y | N | Y | Y | Y | Y | Y | Y | Y | 8/9 |
| Nelson et al. (2006) | Y | N | N | Y | Y | Y | Y | Y | NR | 6/9 |
| Nordwall et al. (2006) | Y | N | N | Y | Y | Y | Y | Y | Y | 7/9 |
| Ogugua et al. (2019) | Y | N | N | Y | Y | Y | Y | Y | NR | 6/9 |
| Osman et al. (2013) | Y | N | N | Y | Y | Y | Y | Y | NR | 6/9 |
| Ou et al. (2017) | Y | N | Y | Y | Y | Y | Y | Y | Y | 8/9 |
| Piccoli et al. (2025) | Y | N | N | Y | Y | Y | Y | Y | Y | 7/9 |
| Rasmussen et al. (2023) | Y | N | N | Y | Y | Y | Y | Y | Y | 7/9 |
| Ruhayel et al. (2010) | Y | N | N | Y | Y | Y | Y | Y | NR | 6/9 |
| Sandhu et al. (2020) | Y | Y | Y | Y | Y | Y | Y | Y | NR | 8/9 |
| Shah et al. (2019) | Y | Y | N | Y | Y | Y | Y | Y | Y | 8/9 |
| Shield et al. (2009) | Y | N | N | Y | Y | Y | Y | Y | Y | 7/9 |
| Singh et al. (2021) | Y | N | N | Y | Y | Y | Y | Y | Y | 7/9 |
| Singh et al. (2022) | Y | N | N | Y | Y | Y | Y | Y | Y | 7/9 |
| Son et al. (2015) | Y | N | N | Y | Y | Y | Y | Y | NR | 6/9 |
| TODAY study group (2021) | Y | Y | N | Y | Y | Y | Y | Y | Y | 8/9 |
| Toopchizadeh et al. (2016) | Y | N | N | Y | Y | Y | Y | Y | Y | 7/9 |
| Unnikrishnan et al. (2008) | Y | N | Y | Y | Y | Y | Y | Y | NR | 7/9 |
| Varley et al. (2022) | Y | Y | Y | Y | Y | Y | Y | Y | NR | 8/9 |
| Verkauskiene et al. (2016) | Y | N | Y | Y | Y | Y | Y | Y | NR | 7/9 |
| Yajaira et al. (2012) | Y | N | Y | Y | Y | Y | Y | Y | NR | 7/9 |
| Walter-Höliner et al. (2018) | Y | N | N | Y | Y | Y | Y | Y | NR | 6/9 |
| Winter et al. (2024) | Y | Y | Y | Y | Y | Y | Y | Y | Y | 9/9 |
| **Abbreviations**: N, no; NA, not applicable; NR, not reported; Y, yes. | | | | | | | | | | |
| **Items of the Joanna Briggs Institute (JBI) Appraisal Checklist for Cohort studies**: | | | | | | | | | | |
| 1. Was the sample frame appropriate to address the target population? | | | | | | | | | | |
| 2. Were study participants sampled in an appropriate way? | | | | | | | | | | |
| 3. Was the sample size adequate? | | | | | | | | | | |
| 4. Were the study subjects and the setting described in detail? | | | | | | | | | | |
| 5. Was the data analysis conducted with sufficient coverage of the identified sample? | | | | | | | | | | |
| 6. Were valid methods used for the identification of the condition? | | | | | | | | | | |
| 7. Was the condition measured in a standard, reliable way for all participants? | | | | | | | | | | |
| 8. Was there appropriate statistical analysis? | | | | | | | | | | |
| 9. Was the response rate adequate, and if not, was the low response rate managed appropriately? | | | | | | | | | | |

# **Figure S1.** Forest plot showing separate prevalence estimates of peripheral neuropathy in children and adolescents with type 1 diabetes and those with type 2 diabetes.

# **Figure S2.** Forest plot showing separate prevalence estimates of autonomic neuropathy in children and adolescents with type 1 diabetes and those with type 2 diabetes.

# **Figure S3.** Forest plot showing separate prevalence estimates of retinopathy in children and adolescents with type 1 diabetes and those with type 2 diabetes.

# **Figure S4.** Forest plot showing separate prevalence estimates of nephropathy in children and adolescents with type 1 diabetes and those with type 2 diabetes.

# **Figure S5.** Forest plot showing separate prevalence estimates of peripheral neuropathy in children and adolescents with type 1 diabetes according to country income.

# **Table S4.** Results of random-effects meta-regressions for the prevalence of microvascular complications in children and adolescents with type 1 diabetes.

| **Outcome (Population)** | **Moderator (Predictor)** | **k (Studies)** | **β Coefficient** | **95% CI** | **p-value** | **R² (%)** |
| --- | --- | --- | --- | --- | --- | --- |
| Nephropathy | Mean HbA1c (%) | 25 | 0.00 | -0.02 to 0.03 | 0.690 | 0.00 |
|  | Years since diagnosis | 21 | -0.00 | -0.02 to 0.01 | 0.914 | 0.00 |
|  | Insulin dose (units/kg/day) | 5 | 0.21 | -0.1 to 0.5 | 0.114 | 99.98 |
|  | Mean age (years) | 19 | 0.00 | -.015 to 0.01 | 0.996 | 0.00 |
|  | Total cholesterol (mg/dL) | 9 | 0.00 | 0.00 to 0.00 | 0.100 | 36.22 |
|  | LDL cholesterol (mg/dL) | 7 | 0.00 | 0.00 to 0.00 | 0.333 | 16.06 |
|  | Cohort year | 19 | -0.00 | -0.01 to 0.00 | 0.381 | 0.00 |
| Retinopathy | Mean HbA1c (%) | 12 | 0.00 | -0.04 to 0.04 | 0.950 | 0.00 |
|  | Years since diagnosis | 13 | 0.01 | -0.02 to 0.04 | 0.592 | 0.00 |
|  | Insulin dose (units/kg/day) | 3 | -0.07 | -0.35 to 0.20 | 0.602 | 0.00 |
|  | Mean age (years) |  |  |  |  |  |
|  | Total cholesterol (mg/dL) | 4 | -0.00 | -.00 to .00 | 0.845 | 0.00 |
|  | LDL cholesterol (mg/dL) | 4 | 0.00 | -0.01 to 0.01 | 0.969 | 0.00 |
|  | Cohort year | 17 | -0.00 | -0.02 to 0.01 | 0.975 | 0.00 |
| Peripheral neuropathy | Mean HbA1c (%) | 26 | -0.02 | -.09 to 0.05 | 0.554 | 0.00 |
|  | Years since diagnosis | 26 | 0.02 | -.024 to 0.07 | 0.354 | 0.00 |
|  | Insulin dose (units/kg/day) | 6 | -0.21 | -1.21 to 0.78 | 0.673 | 0.00 |
|  | Mean age (years) | 30 | -0.01 | -0.04 to 0.02 | 0.409 | 0.00 |
|  | Total cholesterol (mg/dL) | 5 | -0.00 | 0.00 to 0.00 | 0.401 | 0.00 |
|  | LDL cholesterol (mg/dL) | 6 | -0.00 | -0.01 to 0.00 | 0.051 | 32.87 |
|  | Cohort year | 27 | 0.01 | -0.00 to 0.02 | 0.139 | 5.69 |
| Autonomic neuropathy | Mean HbA1c (%) | 11 | 0.03 | -0.18 to 0.25 | 0.748 | 0.00 |
|  | Years since diagnosis | 9 | -0.02 | -0.13 to 0.10 | 0.758 | 0.00 |
|  | Insulin dose (units/kg/day) | 4 | -0.41 | -2.65 to 1.83 | 0.719 | 0.00 |
|  | Mean age (years) |  |  |  |  |  |
|  | Total cholesterol (mg/dL) | 3 | 0.00 | -.00 to 0.01 | 0.554 | 0.00 |
|  | LDL cholesterol (mg/dL) | 4 | 0.00 | -0.00 to 0.01 | 0.235 | 0.00 |

# **Table S5.** Results of random-effects meta-regressions for the prevalence of microvascular complications in children and adolescents with type 2 diabetes.

| **Outcome (Population)** | **Moderator (Predictor)** | **k (Studies)** | **β Coefficient** | **95% CI** | **p-value** | **R² (%)** |
| --- | --- | --- | --- | --- | --- | --- |
| Nephropathy | Mean HbA1c (%) | 11 | -0.00 | -0.04 to 0.03 | 0.803 | 99.79 |
|  | Years since diagnosis | 5 | -0.05 | -0.24 to 0.14 | 0.605 | 0.00 |
|  | Mean age (years) | 10 | -0.02 | -0.06 to 0.02 | 0.293 | 0.00 |
|  | Total cholesterol (mg/dL) | 6 | 0.00 | -0.00 to 0.00 | 0.350 | 7.98 |
|  | LDL cholesterol (mg/dL) | 5 | 0.00 | -0.00 to 0.00 | 0.530 | 33.93 |
|  | Cohort year | 10 | –0.00 | –0.01 to 0.00 | 0.217 | 43.31 |
| Retinopathy | Mean HbA1c (%) | 6 | 0.03 | -0.08 to 0.14 | 0.603 | 0.00 |
|  | Mean age (years) | 5 | -0.00 | -0.08 to 0.08 | 0.968 | 0.00 |
| Peripheral neuropathy | Mean HbA1c (%) | 5 | -0.06 | -0.17 to 0.04 | 0.219 | 1.00 |

# **Figure S5.** Leave-one-out sensitivity analysis for prevalence of peripheral neuropathy in type 1 diabetes.

# **Figure S6.** Leave-one-out sensitivity analysis for prevalence of autonomic neuropathy in type 1 diabetes.

# **Figure S7.** Leave-one-out sensitivity analysis for prevalence of retinopathy in type 1 diabetes.

# **Figure S8.** Leave-one-out sensitivity analysis for prevalence of nephropathy in type 1 diabetes.

# **Figure S9.** Leave-one-out sensitivity analysis for prevalence of peripheral neuropathy in type 2 diabetes.

# **Figure S10.** Leave-one-out sensitivity analysis for prevalence of autonomic neuropathy in type 2 diabetes.

# **Figure S11.** Leave-one-out sensitivity analysis for prevalence of retinopathy in type 2 diabetes.

# **Figure S12.** Leave-one-out sensitivity analysis for prevalence of nephropathy in type 2 diabetes.

# **Figure S13**. Doi plots for peripheral neuropathy in type 1 diabetes patients.

# **Figure S14.** Doi plots for peripheral neuropathy in type 2 diabetes patients.

# **Figure S15.** Doi plots for autonomic neuropathy in type 1 diabetes patients.

# **Figure S16.** Doi plots for retinopathy in type 1 diabetes patients.

# **Figure S17.** Doi plots for retinopathy in type 2 diabetes patients.

# **Figure S18.** Doi plots for nephropathy in type 1 diabetes patients.

# **Figure S19.** Doi plots for nephropathy in type 2 diabetes patients.
